# Bv8/prokineticin 2 is involved in Aβ-induced neurotoxicity

**DOI:** 10.1038/srep15301

**Published:** 2015-10-19

**Authors:** Cinzia Severini, Roberta Lattanzi, Daniela Maftei, Veronica Marconi, Maria Teresa Ciotti, Pamela Petrocchi Passeri, Fulvio Florenzano, Ester Del Duca, Silvia Caioli, Cristina Zona, Gianfranco Balboni, Severo Salvadori, Robert Nisticò, Lucia Negri

**Affiliations:** 1 Institute of Cell Biology and Neurobiology, CNR, Via del Fosso di Fiorano, 64, 00143, Roma; 2 European Brain Research Institute, Via del Fosso di Fiorano, 64, 00143 Rome; 3 Department of Human Physiology and Pharmacology “Vittorio Erspamer”, University of Roma “La Sapienza”, P.za A. Moro 5, 00185 Roma; 4 University of Rome “Tor Vergata” 00133 Rome; 5 IRCCS Fondazione Santa Lucia, Via Ardeatina, 306, 00142 Roma; 6 Department of Life and Environmental Sciences, University of Cagliari, Via Ospedale, 72, 09124 Cagliari; 7 Department of Pharmaceutical Sciences, Università of Ferrara, Ferrara

## Abstract

Bv8/Prokineticin 2 (PROK2) is a bioactive peptide initially discovered as a regulator of gastrointestinal motility. Among multiple biological roles demonstrated for PROK2, it was recently established that PROK2 is an insult-inducible endangering mediator for cerebral damage. Aim of the present study was to evaluate the PROK2 and its receptors’ potential involvement in amyloid beta (Aβ) neurotoxicity, a hallmark of Alzheimer’s disease (AD) and various forms of traumatic brain injury (TBI). Analyzing primary cortical cultures (CNs) and cortex and hippocampus from Aβ treated rats, we found that PROK2 and its receptors PKR_1_ and PKR_2_ mRNA are up-regulated by Aβ, suggesting their potential involvement in AD. Hence we evaluated if impairing the prokineticin system activation might have protective effect against neuronal death induced by Aβ. We found that a PKR antagonist concentration-dependently protects CNs against Aβ_1–42_-induced neurotoxicity, by reducing the Aβ-induced PROK2 neuronal up-regulation. Moreover, the antagonist completely rescued LTP impairment in hippocampal slices from 6 month-old Tg2576 AD mice without affecting basal synaptic transmission and paired pulse-facilitation paradigms. These results indicate that PROK2 plays a role in cerebral amyloidosis and that PROK2 antagonists may represent a new approach for ameliorating the defining pathology of AD.

Alzheimer’s disease (AD) is an irreversible/chronic progressive neurodegenerative disease, characterized by extracellular deposition of Aβ plaques and intracellular accumulation of hyper-phosphorylated tau protein in neurofibrillary tangles^1^.

Substantial evidence indicates that Aβ plaque processes might be the central players in AD pathology^2^,^3^. Senile plaques are intimately surrounded by morphologically abnormal dendrites and axons and are infiltrated by astrocytes and microglia in and around their central amyloid core^4^,^5^. Once activated, astrocytes and microglia produce several pro-inflammatory signal molecules, including cytokines, growth factors, complement molecules, cell adhesion molecules and chemokines^6^. This activation is thought to result from the glial reaction to the events related to the ongoing deposition of Aβ^7^,^8^, leading to an inflammatory hypothesis^6^. Indeed, analysis of human brain AD samples has revealed highly expressed inflammatory cytokines during the early stages of AD, and genome-wide studies showed an up-regulation of inflammatory genes, indicating a potential role of inflammation in the progression of AD^9^.

Chemokines are a group of cytokines originally identified as factors regulating the migration of leukocytes in inflammatory and immune responses^10^. While it has been reported that chemokines exert physiological actions in the healthy brain^11^, they have been shown to be produced under various pathological conditions including AD^12^,^13^. To confirm these data, several chemokines and chemokine receptors have been found to be up-regulated in the AD brain^14^.

A new family of chemokines, the Bv8/Prokineticin family has recently emerged as a critical player in immune system and inflammatory diseases. They are secreted bioactive peptides highly conserved across species^15^,^16^. In mammals, this family consists of two ligands: EG-VEGF/prokineticin1 (PROK1) and mammalian-Bv8/PROK2 and of two G-protein coupled receptors: PKR_1_ and PKR_2_. The amphibian homologue, Bv8, isolated from the skin secretion of the frog *Bombina variegata* displayed pharmacological activity like the mammalian molecule PROK2, with comparable affinity for both receptors^17^. Bv8 also represents a good pharmacological tool to study the effect of PROK2 *in vitro* and *in vivo*, as we have already demonstrated^18^,^19^,^20^,^21^,^22^,^23^,^24^,^25^,^26^. Since their discovery, multiple physiological roles for the prokineticins have been evidenced. Accordingly, they were shown to promote angiogenesis and modulate neurogenesis, circadian rhythms, hematopoiesis, and immune response^19^.

The prokineticin receptors, PKR_1_ and PKR_2_ are localized in the brain, dorsal root ganglia (DRG) neurons, granulocytes, macrophages, and endothelial cells. The agonist PROK2 is expressed in discrete nuclei into the brain^20^ and is constitutively expressed, at very low levels, in some DRG neurons^21^,^22^, in bone marrow, spleen and in peripheral blood cells but it is strongly up-regulated in inflammatory diseases and tumours, associated with infiltrating cells^18^,^23^,^24^. We have recently demonstrated that peripheral nerve injury causes a dramatic increase of PROK2 in DRG neurons and in activated astrocytes in the spinal cord, associated with development of neuropathic pain^22^ and identified PROK2 as a critical mediator of experimental autoimmune encephalomyelitis (EAE), animal model of Multiple Sclerosis^25^.

Recently, it was demonstrated that PROK2 is an insult-inducible endangering mediator for cerebral ischemic injury, identifying this bioactive peptide as a potential therapeutic target for stroke, supporting a role in brain pathological states^26^. Given that mRNA PROK2 expression is up-regulated by several pathological stressors, including hypoxia, reactive oxygen species, and excitotoxic glutamate, here we investigated if Aβ, the central player in AD, might induce a pathological condition leading to over-expression of the prokineticin system.

To this aim, we examined the expression profile of PROK2, PKR_1_ and PKR_2_ at basal conditions and after Aβ insult *in vitro*, in primary cortical cultures, and *in vivo*, in a non-transgenic rat model of AD obtained by intracerebroventricular (i.c.v.) injection of Aβ.

Furthermore, we investigated the potential neuroprotective effect resulting from the pharmacological blockade of the prokineticin system. Our data suggest that Aβ induces activation of the prokineticin system which may represent a new pathological hallmarks in animal models and possibly a novel therapeutic target for AD.

## Results

### Aβ-induced neuronal apoptosis in primary cortical cultures

**Figure 1:**
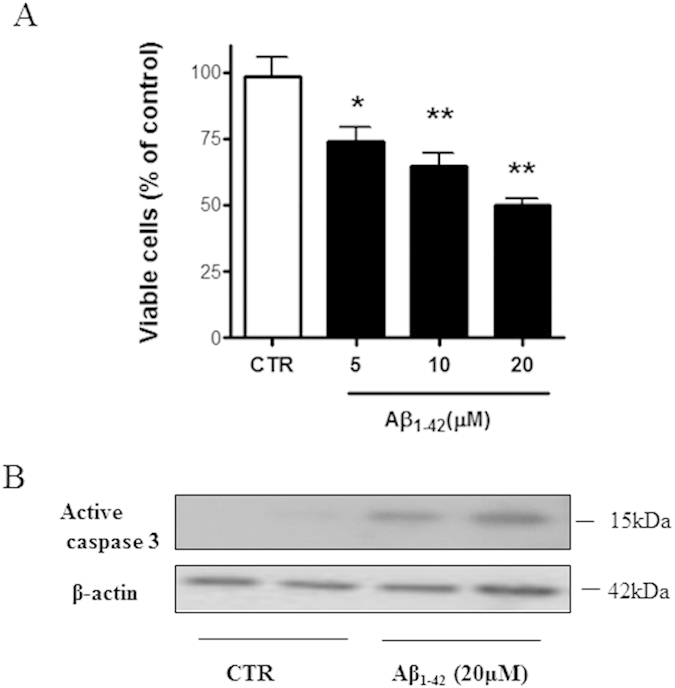
(**A**) Aβ_1–42_ -induced neurotoxicity CNs (1 × 10^6^ cells/well) were treated at 12 DIV with different Aβ_1–42_ concentrations (5, 10, 20 μM) and then assayed for cell viability 48 h later. Data represent mean (±SEM) from at least 4 independent experiments run in duplicate. Statistically significant differences were calculated by one-way analysis of variance (ANOVA) for repeated measures followed by Bonferroni’s test for multiple comparisons (*p < 0.05, **p < 0.01 vs CTR). (**B**) Caspase 3 involvement in Aβ_1–42_ -induced apoptosis Immunoreactive signal of 17/19 kDa caspase 3 active fragment (Asp 175) from control and Aβ_1–42_ (20 μM) treated cells, normalized against β_−_actin, run in duplicate. Molecular mass markers (kDa) are shown on the right.

Incubation of cortical cultures (CNs) with Aβ_1–42_ for 48 h reduced cell survival in a concentration-dependent manner. As shown in Fig. 1A, 20 μM of Aβ_1–42_ induced about 50% reduction in cells viability and this concentration was chosen to investigate a possible involvement of the prokineticin system in Aβ-induced toxicity. To characterize the identity of the dying cells, we quantified the number of dying neurons (NeuN-positive cells) and of dying astrocytes (GFAP-positive cells) following Aβ_1–42_ treatment (20 μM). The amount of NeuN^+^ cells decreased of about 50% (710 ± 95 in control group vs 290 ± 31 in Aβ treated cultures) while number of GFAP^+^ cells was not affected (560 ± 65 in control group vs 522 ± 87 in Aβ treated cultures), thus demonstrating that Aβ toxicity is mainly directed to neurons. To confirm that Aβ exerts its toxicity through an apoptotic mechanism, we analyzed, by Western blot analysis, the appearance of the caspase 3 active fragment. As shown in Fig. 1B, following Aβ_1–42_ treatment, this fragment was detected.

### Up-regulation of PROK2, PKR_1_ and PKR_2_ expression by Aβ insult

Using qRT-PCR, we examined mRNA expression levels of PROK2 and of its receptors PKR_1_ and PKR_2_ in primary cortical cultures treated with Aβ_1–42_ (20 μM) at different time-points. Time-course analysis (6, 12, 24 h) indicated that PROK2 mRNA was significantly increased after 6 h Aβ_1–42_ treatment, and returned to basal levels at 12 and 24 h.

**Figure 2:**
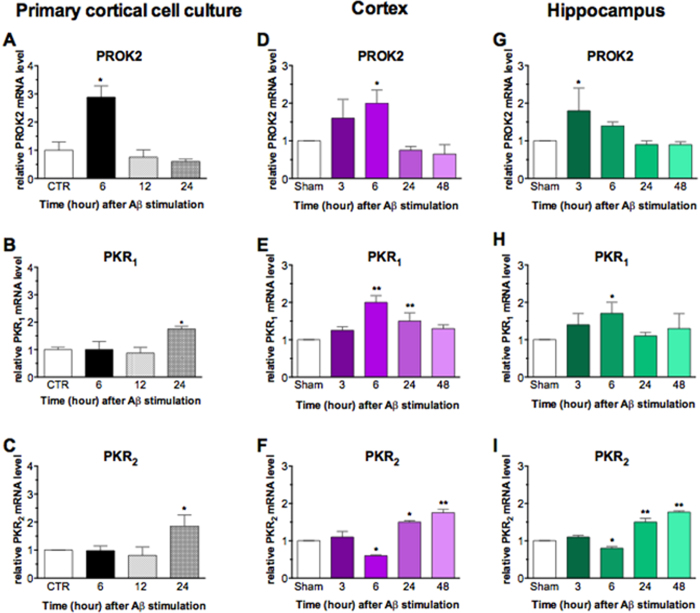
Aβ_1–42_ effect on PROK2, PKR_1_ and PKR_2_ mRNA. Relative PROK2, PKR_1_ and PKR_2_ mRNA levels were determined by qRT-PCR in CNs (1 × 10^6^ cells/well) treated at 12 DIV with Aβ_1–42_ (20 μM) for different times (**A**–**C**), in cortex (**D**–**F**) and hippocampus (**G**–**I**) from Aβ_1–42_ injected rats at different time points. The mRNA expression levels were expressed in relation to β-actina and presented as fold of increase relative to control cultures or Sham rats. Data represent means (±SEM) from at least 4 independent experimental points run in triplicate and statistically significant differences were calculated by one-way analysis of variance (ANOVA) for repeated measures followed by Bonferroni’s test for multiple comparisons (*p < 0.05, **p < 0.01 vs CTR or sham).

Both PKR_1_ and PKR_2_ mRNA were considerably increased after 24 h treatment (Fig. 2A–C). In order to assure about measuring significant levels of transcript we report the Ct values (mean ± SEM, 5 samples) for vehicle-treated cultures: PROK2 28.1 ± 0.3; PKR_1_ 32.5 ± 0.8; PKR_2_ 31.6 ± 0.6.

Next, we examined the expression levels of PROK2 and its receptors in rat cortex and hippocampus 3, 6, 24 and 48 h after i.c.v. injection of Aβ_1–42._ In the cortex, time-course analysis indicated about two-fold increase in PROK2 expression after 6 h (Fig. 2D). PKR_1_ mRNA was significantly increased after 6 and 24 h, whereas PKR_2_ mRNA showed a biphasic pattern of expression, with a decrease at 6 h followed by an increase at 24 and 48 h (Fig. 2E,F). In hippocampus, PROK2 expression increased earlier, showing a peak at 3 h after Aβ_1–42_ injection (Fig. 2G). The Ct values in control animals (5 animals/group) were: in the cortex: 32.1 ± 0.2; 31.9 ± 0.7; 32.4 ± 0.6; 31.8 ± 0.4; 31.7 ± 0.3 at time 0, 3, 6, 24 and 48 h; in hippocampus: 32.8 ± 0.4; 32.1 ± 0.3; 32.6 ± 0.5; 31.9 ± 0.6; 32.1 ± 0.7 at time 0, 3, 6, 24 and 48 h.

The expression of PKR_1_ and PKR_2_ showed a similar pattern in hippocampus as in cerebral cortex, with an increase of PKR_1_ at 6 h and a biphasic pattern of expression for PKR_2_, with a decrease at 6 h followed by an increase at 24 and 48 h (Fig. 2H,L). The Ct values in control animals (5 animals/group) were: in the cortex PKR_1_ 34.8 ± 0.3; 34.1 ± 1.4; 35.3 ± 0.4; 35.8 ± 0.7; 34.5 ± 0.5 and PKR_2_ 31.7 ± 0.2; 31.8 ± 1.5; 31.3 ± 0.9; 32.3 ± 0,6; 32.6 ± 0.4. In hippocampus PKR_1_ 34.4 ± 0.5; 34.3 ± 0.4; 34.9 ± 0.3; 34.1 ± 0.7; 34.2 ± 0.8 and PKR_2_ 32.5 ± 0.3; 32.3 ± 0.4; 31.9 ± 0.8; 32.5 ± 0.6; 32.3 ± 0.3 at time 0, 3, 6, 24 and 48 h.

The reported Ct values indicate that the constitutive basal expression of PKR_2_ is always higher than that of PKR_1_.

### Localization of PROK2 and its receptors in CNs after Aβ treatment

**Figure 3:**
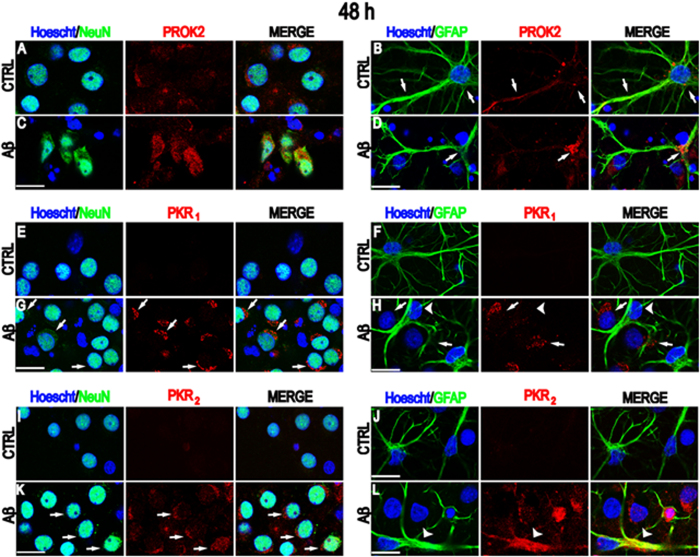
PROK2, PKR_1_ and PKR_2_ protein localization in control conditions and after Aβ_1–42_ treatment. Representative confocal images of cultured mixed cortical neurons (CNs) stained with anti-PROK2 (**A**–**D**) or anti PKR_1_ (**E**–**H**) or anti PKR2 antibodies (**I**–**L**) (red) in control conditions (CTRL) and after 48 h Aβ_1–42_ treatment (Aβ). Neurons were stained with NeuN (green), astrocytes with GFAP (green), and nuclei with Hoecsht (blue). Panel (**A–D**) PROK2 low to medium immunoreactivity was found in neuronal cell bodies and astrocytes processes (arrows) in control conditions (first row) which increased after Aβ_1–42_ treatment mainly in the cell bodies (second row: arrow). Panel (**E–H**) PKR_1_ immunoreactivity was undetectable (first row) under control conditions in both neurons and astrocytes. After Aβ_1–42_ treatment PKR_1_ immunoreactivity displayed a selective neuronal increase confined to putative endoplasmic reticulus-Golgi complex (arrows) while astrocytes were still devoid of staining (arrowheads). Please note, close to astrocytes there are some PKR_1_ positive neurons (arrows). Panel (**I–L**) Very low PKR_2_ immunoreactivity was detected under control conditions while, after Aβ_1–42_ treatment increased in neuronal cell bodies and astrocyte processes (arrows). Note the selective increase in a dying cell body surrounded by astrocytic processes (arrowheads). Scale bar: 15 μm.

By immunofluorescence studies we looked for protein localization of PROK2 and its PKRs on neurons and astrocytes of primary mixed cortical cultures. Under control conditions, PROK2 immunoreactivity is present in body neurons (NeuN + cells) and in astrocytes processes (GFAP+ cells) (Fig. 3A,B), while PKR_1_ and PKR_2_ immunoreactivity were virtually undetectable (Fig. 3E–J). 12 h, 24 h and 48 h Aβ_1–42_ treatment time-dependently increased PROK2 in both neurons and astrocytes (Additional Fig. 1 and 3). After 48 h treatment PROK2 immunoreactivity was localized mainly into cell bodies (Fig. 3C,D). Time discrepancy between PROK2 mRNA (6 h) and protein (24–48 h) maximum increase might depend on accumulation of the synthesized protein into cortical neurons and astrocytes.

Conversely, PKR_1_ immunoreactivity appeared specifically increased in neuron (arrows) but not in astrocyte (arrowhead) cell bodies and localized to perinuclear structures resembling the endoplasmic-Golgi membranes complex (Fig. 3G,H). PKR_2_ immunoreactivity appeared increased and localized to both neuronal cell bodies and astrocytes (Fig. 3K,L), with a diffuse vesiculate cytoplasmatic pattern (arrows).

### Effects of PC1, a PKRs antagonist, on Aβ-induced toxicity

**Figure 4:**
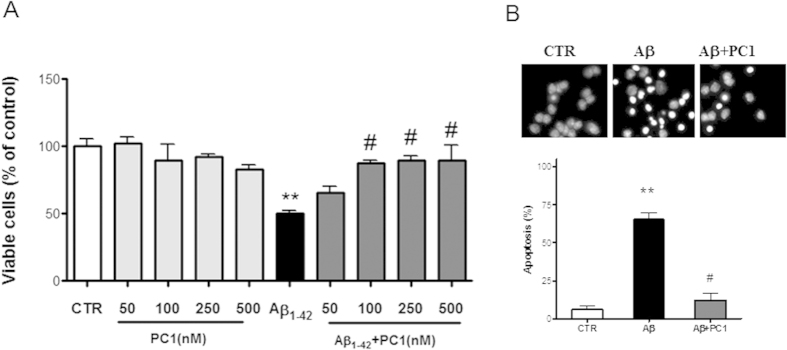
(**A**) Effect of PC1 against Aβ neurotoxicity CNs (1 × 10^6^ cells/well) were treated at 12 DIV with Aβ_1–42_ (20 μM) alone or in the presence of increasing concentrations of PC1 (50, 100, 250, 500 nM) and then assayed for cell viability 48 h later. Data from the same concentrations of PC1 alone are also shown. Data represent mean (±SEM) from at least 4 independent experiments run in duplicate. (**B**) Protective effect of PC1 on nuclear condensation induced by Aβ_1–42_ in terms of Hoechst staining Representative immunofluorescence photomicrographs showing cortical cells stained with Hoechst in control conditions (CTR), after 48 h incubation with Aβ_1–42_ (20 μM) (Aβ), simultaneously exposed to Aβ_1–42_ and PC1 (100 nM) for 48 h. Histogram shows quantification of Hoechst-stained (apoptotic) neurons for each treatment. Five fields were selected for each treatment from three independent experiments (n = 3). Data represent means (±SEM) from at least 4 independent experiments run in duplicate and statistically significant differences were calculated by one-way analysis of variance (ANOVA) for repeated measures followed by Bonferroni’s test for multiple comparisons (**p < 0.01 versus CTR; #p < 0.01 versus Aβ_1–42_).

Given that Aβ exposure clearly induced activation of the prokineticin system, we tested the activity of PC1, a non-peptide Bv8 antagonist, against Aβ_1–42_ toxicity in primary cortical cultures (CNs). Different concentrations of PC1 were co-applied with Aβ_1–42_ (20 μM) to CNs for 48 h. Quantitative analysis revealed that incubation of CNs with Aβ_1–42_ for 48 h caused ~ 50% reduction in the number of surviving cells, as compared to vehicle treated cells (controls, CTR). PC1 (50, 100, 250 and 500 nM) dose-dependently reversed the Aβ_1–42_ toxicity, inducing 15.5 ± 5.0%, 37.6 ± 1.9%, 39.5 ± 3.2 and 39.6 ± 11.3% increase in cell viability, as compared to Aβ condition (Fig. 4A).

The results obtained were confirmed by cell viability quantification by Hoechst staining, indicating the same percentage acquired counting intact nuclei. As shown in Fig. 4B, Aβ_1–42_ (20 μM) induced apoptotic cell death with nuclear chromatin condensation or fragmentation and this effect was suppressed by PC1 (100 nM) treatment.

**Figure 5:**
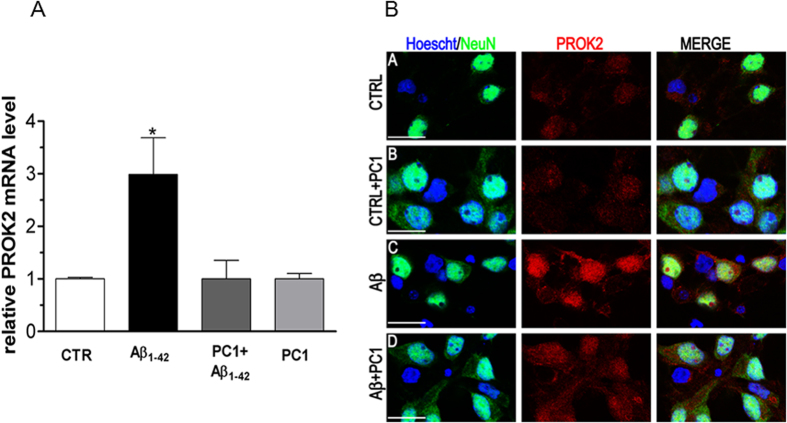
PC1 effect on PROK2 up-regulation. (**A**) Relative PROK2 mRNA levels were determined by qRT-PCR in CNs (1 × 10^6^ cells/well) treated for 6 h with Aβ_1–42_ (20 μM), PC1 (100 nM), alone or in combination. The mRNA expression levels were expressed in relation to b-actina and presented as fold of increase relative to controls (controls Ct: 28.3 ± 0.1. Data represent means (±SEM) from at least 2 independent experiments run in triplicate and statistically significant differences were calculated by one-way analysis of variance (ANOVA) for repeated measures followed by Bonferroni’s test for multiple comparisons (*p < 0.05 vs CTR). (**B**) Representative immunofluorescence photomicrographs of cultured mixed cortical neurons (CNs) stained with anti-PROK2 antibody (red) in control conditions (CTRL) and after 48 h Aβ_1–42_ treatment (Aβ) alone or in the presence of PC1 (200 nM). Neurons were stained with NeuN (green), and nuclei with Hoecsht (blue). Note the cytoplasmatic increase in PROK2 which is reversed by PC1 treatment. Scale bar: 15 μm.

It was previously reported that PC1 is able to reduce PROK2 up-regulation in DRG neurons and in activated astrocytes of spinal cord following nerve injury^22^,^27^. qRT-PCR experiments demonstrated, also in this system, that co-incubation with Aβ_1–42_ and PC1 (100 nM) prevented the Aβ-induced PROK2 mRNA up-regulation (Fig. 5A). To confirm these data at protein level, we performed immunofluorescence studies looking for the localization of PROK2 in CNs treated with PC1 alone or in the presence of Aβ for 48 h. As shown in Fig. 5B, PROK2 immunofluorescence was clearly higher in Aβ-treated than in PC1/Aβ-treated neurons.

### Neurotoxic activity of Bv8 (PROK2)

**Figure 6:**
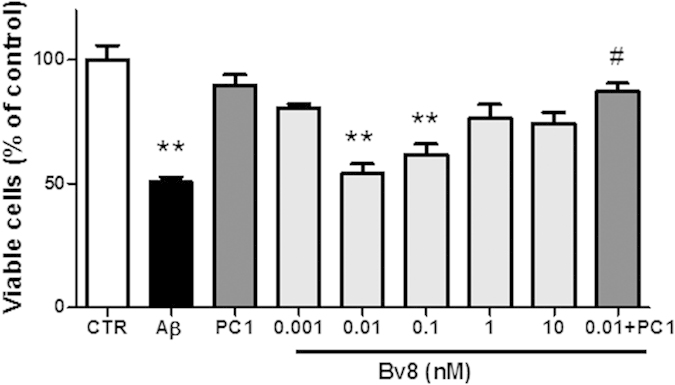
Effect of Bv8. CNs (1 × 10^6^ cells/well) were treated at 12 DIV with different concentrations of Bv8, alone or in the presence of PC1 (100 nM) and then assayed for cell viability 48 h later. Data from the corresponding concentrations of PC1 alone are also shown. Data represent mean (±SEM) from at least 3 independent experiments run in duplicate. Statistically significant differences were calculated by one-way analysis of variance (ANOVA) for repeated measures followed by Bonferroni’s test for multiple comparisons (**p < 0.01 vs CTR; #p < 0.01 ves Bv8 0.01 nM).

The antagonistic effect of PC1 on Aβ-induced toxicity led us to hypothesize that prokineticins could exert a toxic activity on CNs. To this aim, CNs were treated with different concentrations of Bv8 for 48 h. Quantitative analysis revealed that Bv8 (0.001, 0.01, 0.1, 1 and 10 nM) dose-dependently reduced cell viability, inducing 19.5 ± 1.6%, 46 ± 4%, 38.5 ± 4.4, 24 ± 5.7 and 26 ± 4.4% reduction of viable cells, as compared to control conditions (Fig. 6). To confirm the activity of PC1 on its own agonist, we have co-incubated CNs with PC1 (100 nM) and the most effective concentration of Bv8 (0.01 nM). As shown in the same figure, PC1 completely reversed Bv8 toxic activity.

### PC1 prevents LTP impairment in Tg2576 hippocampal slices

Previous studies from our group^28^ and others^29^ have shown that the magnitude of LTP, induced with a high-frequency stimulation protocol, is impaired in the hippocampus of adult Tg2576 (TG) mouse model of AD compared with age-matched wild-type (WT) controls.

**Figure 7:**
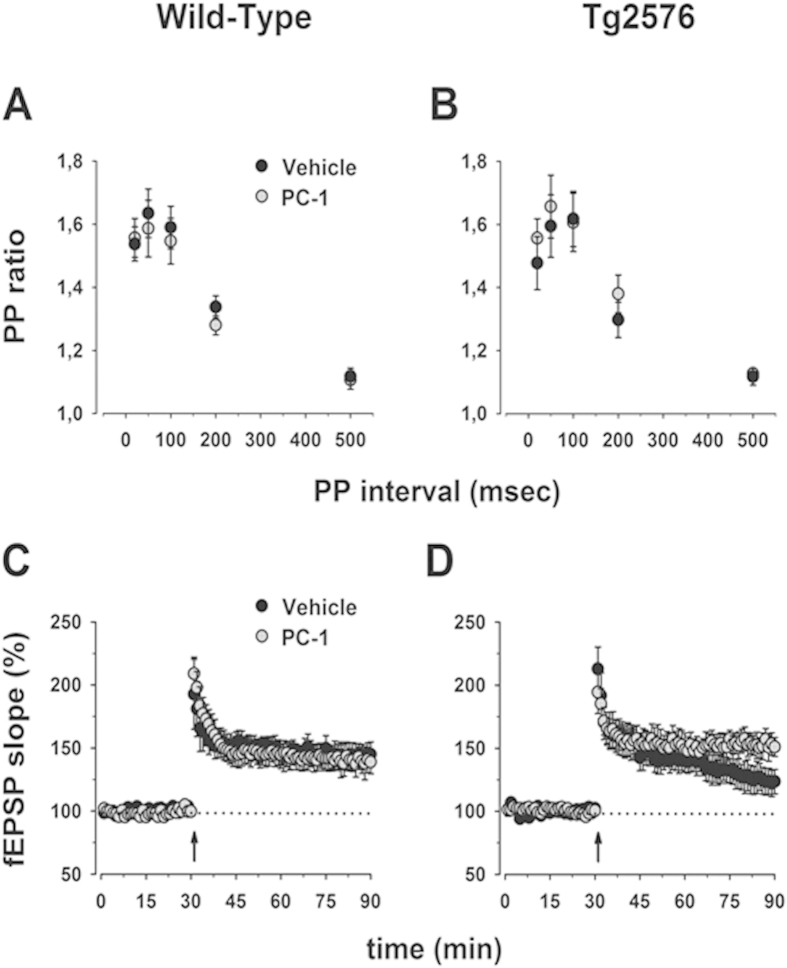
Effect of PC-1 on LTP impairment in Tg2576 mice. Paired pulse ratios (slope of second EPSP/slope of first EPSP) were plotted as a function of the inter-pulse interval for 6–9 month old non-transgenic (panel **A**) and Tg2576 (panel **B**) mice. At least 11 slices from 8 different mice are shown for each experimental condition. No significant differences between non-transgenic and Tg2576 mice, and between treatment groups were detected. Superimposed pooled data representing normalized changes in the field potential slope (±SEM) induced by HFS (100 Hz, 1 s) are shown for non-transgenic (panel (**C)**) and Tg2576 (panel (**D)**) mice. At least 7 slices from 7 different mice are shown for each experimental condition. A significant reduction of LTP in Tg2576 compared to non-transgenic mice was observed (*p* < 0.05). Perfusion with PC-1 (50 nM) was able to rescue LTP impairment in Tg2576 mice (*p* < 0.05), but had no effect in slices from wild-type controls. PC-1 was continuously bath applied to hippocampal slices throughout the experiment.

Here we recorded field excitatory post-synaptic potentials (fEPSPs) from the *stratum radiatum* of the CA1 area upon stimulation of the Shaffer collaterals pathway every 30 s, a test stimulation intensity attaining a half-maximal response. In agreement with our previous report indicating a similar efficacy of the basal synaptic transmission in WT and TG mice^28^, the input-output curves in WT and TG slices was not significantly different (data not shown). Similarly, the paired-pulse facilitation (PPF) paradigm, a presynaptically mediated short-term enhancement of transmission, was unaffected in all the conditions tested (*p* > 0.05, Fig. 7A,B).

However, LTP produced by high-frequency stimulation (100 Hz, 1 s), was decreased in TG mice (123 ± 10) when compared to WT controls (145 ± 10) (*p* < 0.05, Fig. 7C,D). To test the hypothesis that pharmacological inhibition of PKR rescues synaptic plasticity impairment, we measured LTP in acute TG hippocampal slices in the presence of the PK receptor antagonist PC1 (50 nM), or vehicle. We found that the degree of potentiation in WT slices (145 ± 10) was independent from the presence of PC1 (139 ± 10) (*p* > 0.05, Fig. 7C), indicating that PK blockade does not affect synaptic plasticity in physiological conditions. Notably, LTP in PC1-treated TG hippocampal slices was significantly rescued (151 ± 8) compared to vehicle-incubated TG slices (123 ± 10) (*p* < 0.05, Fig. 7D), suggesting that inhibition of PK receptors in TG neurons is sufficient to restore synaptic plasticity to WT levels.

### PC1 reverts Aβ-induced toxicity in hippocampal cultures

Since PC1 was able to restore synaptic plasticity in the hippocampus of adult Tg2576 mice, we extended the study of PC1 against Aβ_1–42_ toxicity in hippocampal primary cultures (HNs). Quantitative analysis revealed that incubation of HNs with Aβ_1–42_ (20 μM) for 48 h caused ~ 50% reduction in the number of surviving cells, as compared to control cells (CTR). PC1 (100 nM) significantly reduced the Aβ_1–42_ toxicity, inducing 28.1 ± 2.9% increase in cell viability, as compared to Aβ condition (Additional Fig. 2).

## Discussion

Neuropathological hallmarks of AD correlate with the presence of soluble Aβ oligomers as the principal neurotoxic agent^30^,^31^. Aβ has been so far one of the most important targets for the development of AD drugs^32^,^33^ so, molecules able to counteract Aβ toxicity will be potential therapeutic agents.

It has been proposed that Aβ plaques stimulate a chronic inflammatory reaction^6^. Neuroinflammation clearly occurs in pathologically vulnerable regions of the AD brain, with increased expression of acute phase proteins and proinflammatory mediators, which are hardly evident in the normal brain^5^,^34^,^35^. Indeed, several chemokines and chemokine receptors have been found to be up-regulated in the AD brain^14^.

The study here presented shows that the chemokine prokineticin 2 (PROK2) and its receptors are involved in Aβ toxicity both *in vitro* and *in vivo*. Indeed, our results demonstrated that mRNA and protein levels of PROK2, PKR_1_ and PKR_2_ are significantly modified by Aβ treatment, suggesting that modulation of prokineticin system could be a general response to Aβ injury.

Neurons are not the only cell type in the brain affected in AD; vulnerable brain regions exhibit activated microglial cells and astrocytes, which often associate with amyloid deposits, suggesting a central role of these non-neuronal cells in AD pathology^36^,^37^. Different cell types including neurons may produce chemokines, however, the main neural cells producing chemokines in response to Aβ seem to be astrocytes^38^,^39^,^40^. Accordingly, PROK2 immunoreactivity is significantly increased in response to Aβ, in both neurons and astrocytes. Also the prokineticin receptors, almost absent in control conditions, are increased after Aβ stimulation: PKR_1_ immunoreactivity being mainly increased in neurons and PKR_2_ in both cell types.

The functional involvement of the PK system in Aβ toxicity was further demonstrated by the ability of PC1, a non-peptide antagonist of the prokineticin receptors^41^,^42^,^43^, to prevent Aβ toxicity and by the ability of Bv8, the amphibian homologue of the mammalian PROK2, to induce apoptosis comparable to that induced by Aβ, in cortical brain cultures. The Bv8-inuced apoptosis was prevented by co-administration of PC1, as well.

The here demonstrated pro-apoptotic effect of Bv8 disagrees with previous data showing that Bv8 protects against Oxigen Glucose Deprivation stimulated ischemia (Pellegrini, personal communication) and against NMDA-induced excitotoxicity in murine cortical cultures^44^, and PROK2 protects cardiomyocytes against oxidative stress^45^. It is worthy of note that, whereas the Bv8 protective effects were obtained with nanomolar concentrations (10–100 nM), the Bv8 neurotoxic activity, observed in our experiments, was achieved with picomolar concentrations (0.01 and 0.1 nM), while higher concentrations did not seem to be toxic. Such low concentrations could be compatible with the small amount of PROK2 eventually released by Aβ treatment. PKR_1_ signaling regulates its own ligand expression^18^,^22^,^42^ establishing an autocrine and paracrine loop.

Under normal conditions, activation of prokineticin receptors has been shown to stimulate the Erk1,2/MAPK and Akt pathways^20^,^44^,^46^, which have been mostly associated with cell survival but which also can exert deleterious effect as in ischemia and inflammation. PROK2, such as Bv8, has comparable affinity for either receptors but activates more efficiently the PKR_1_^46^,^47^ which is up-regulated in neurons, too. Hence PROK2, at different concentrations and/or during insult conditions, might activate differential signaling^26^.

Interestingly, in rat embryo of 18 pc, both the receptors are highly expressed in the neuroepithelium lining brain ventricles but the PKR_1_ expression is significantly decreased in brain cortex of neonatal rats, and apparently lacks in brain cortex of adult rats, whereas PKR_2_ is still expressed at high levels in the cortex and other regions of forebrain^48^. This suggests that developmental apoptotic processes hit mainly the PKR_1_ expressing cells. Hence the upregulation of this receptor, induced mainly in neuronal cells by toxic insult, might be responsible for neuronal apoptosis, whereas PKR_2_ might mediate protective signaling, triggered by the protective (10–100 nM) concentrations of Bv8.

Here we demonstrated that PC1 concentration-dependently reversed Aβ toxicity. Previous data acquired with PC1 clearly indicated that it is able to reduce expression and storage of PROK2^22^,^27^ in cells and tissues. This mechanism of action was confirmed at both mRNA and protein level, in here reported experiments demonstrating that the increased amount of PROK2 into neurons is significantly reduced by PC1 treatment. Hence, block of PK receptors by PC1 reduces PROK2 levels, so impairing the pro-apoptotic signaling.

The ability of PC1 to antagonize Aβ toxicity on the one hand, and LTP impairment in the APP Tg2576 mouse model of AD on the other, suggests that injury-induced PROK2 expression is deleterious and that blocking of the prokineticin system may be therapeutic. In line with this, several chemokine signaling molecules are known to modulate cognitive function and synaptic plasticity, either in physiological and pathological conditions^49^,^50^. Accordingly, the selective targeting of the chemokine system has proven beneficial in diseases associated with synaptic dysfunction and memory impairment, including AD^51^. Certainly, the possible effects of PROK2 dysregulation at the synaptic level remain largely unexplored and might involve complex mechanisms. For example, previous electrophysiological experiments have demonstrated that PROK2 might reduce the GABAergic function^52^,^53^ or modulate voltage-gated ion channels^54^, the balance of which finely regulates intrinsic excitability and synaptic plasticity events. Thus, application of PC1 might rescue LTP impairment in the Tg2576 model by preserving synaptic homeostasis and vulnerability to the PROK2 insult.

Taken together, these results indicate that PROK2 plays a role in Aβ-mediated neuronal death both *in vitro* and *in vivo*, representing a new approach in the elucidation of AD etiopathology.

However, further studies will be necessary to assess whether activation of PK signaling indeed is involved in AD and whether PROK2 antagonists could actually be used as a therapeutic strategy.

## Methods

### Chemicals

Aβ_1–42_ was purchased from Abcam (Abcam, Cambridge, UK). Aβ peptide stock solutions at a concentation of 1 mg/ml were prepared in PBS (0.01 M NaH_2_PO_4_, 0.15 M NaCl, pH 7.4) and stored to −20 °C. Aliquots of Aβ peptides were allowed aggregating by incubation at 37 °C for 72 h before *in vivo* infusion^55^. In the Additional Fig. 3, Western blot results showed that Aβ oligomers preparations comprise a mixture of dimers, trimers, and tetramers (from 4 to 20 kDa) and larger oligomers high-molecular-weight (molecular masses ranging from 70 to 100 kDa). All other reagents were also from Sigma (St. Louis, MO, Missouri), if not specifically reported.

### Surgical procedures

All procedures were approved by the Italian Ministry of Health (Rome, Italy) and performed in compliance with the guidelines of the US National Institutes of Health and the Italian Ministry of Health (D.L.116/92). All efforts were made to minimize the number of animals and their suffering.

Male adult Sprague-Dawley rats (Charles River, Como, Italy) weighting 250–275 g were housed in individual plastic cages under optimum light conditions (12:12 h light–dark cycle), temperature (22 ± 2 °C), and humidity (52 ± 2%), with food and water provided *ad libitum*. Under ketamine-xylazine anaesthesia (60 + 10 mg/kg, i.p.), each rat was implanted surgically with a plastic guide cannula (Linca, Tel-Aviv, Israel), stereotaxically inserted through a skull hole drilled over the left lateral ventricle of the brain (1 mm caudal to and 1.8 mm lateral to the bregma). The cannula was screwed into the skull hole and secured to the bone with dental cement. After one week-recovery from surgery, Aβ_1–42_ (1 nmol) or saline solution were intracerebroventricularly (i.c.v.) injected, in a constant volume of 5 μl in awake rats, using a 10-μl Hamilton syringe fitted with a 26-gauge needle that was inserted through the guide cannula to a depth of 4.2 mm below the external surface of the skull. The needle was left in place for 10 s after the end of the injection to avoid reflux of the solute.

### RNA purification, reverse transcription, and RNA determination by quantitative RT-PCR

For the *in vitro* studies, cortical cultures were treated with Aβ_1–42_ (20 μM) for 6, 12 and 24 h. For *in vivo* studies, animals were euthanized 3, 6, 24 and 48 h following Aβ_1–42_ (1 nmol) i.c.v. injection. Total RNA was extracted using the TRIzol solution Invitrogen (Carlsbad, CA, USA), according to the manufacturer’s instructions. For *in vivo* samples, tissues were homogenized using a power homogenizer and insoluble material was removed by centrifugation at 12.000 g for 10 min at 8 °C. To obtain cDNA, 2 μg total RNA was reverse transcribed in MLV reverse transcription buffer (Promega, Madison, WI) containing the following: 40 μg/ml random primers (Promega, Madison, WI), 1 mM dNTP, 40 μg/ml of Recombinant RNasin Ribonuclease Inhibitor (Promega, Madison, WI), and MLV reverse transcriptase (Promega, Madison, WI) in a final volume of 25 μl. The reaction was incubated at 37 °C for 60 min. Messenger RNA expression was quantitatively measured with real time quantitative PCR (ABI Prism 7700 Sequence Detector; Perkin Elmer Applied Biosystems, Foster City, CA) using SYBR Select MasterMix fluorescence (Applied Biosystems).

The primer sequences used in this study were as following: for rat PROK2, forward 5′-TCATCACCGGGGCTTGCG -3′, reverse 5′-TAACTTTCCGAGTCAGGG -3′; for rat PKR1, forward 5′-CGCACCGTCTCCCTCTAC-3 and reverse 5′- GTTTGACACTTCATCCGCG-3′; for rat PKR2, forward 5′-CTCCGTCAACTACCTTCGTA-3′ and reverse 5′-GAGGCGGTCTGGTAATTCA-3′.

The internal reference Tata Binding Protein (TBP) primer was purchased from Invitrogen. Real time PCR amplification and product detection was performed using an ABI PRISM 7900 FAST REAL TIME PCR (Applied Biosystems, Fostercity, CA, USA). Each assay included a standard curve sample in duplicate, a no template control, and the cDNA sample from treated cells in triplicate for each point. For each set of primers, a no template control and a no reverse transcriptase control was included. The thermal cycling conditions were: 95 °C, 2 min for denaturation, followed by 40 cycles of 95 °C, 15 sec, 60 °C, 1 min. Post-amplification dissociation curves were performed to verify the presence of a single amplification product and the absence of genomic DNA contamination. The Ct value of the specific gene of interest was normalized to the Ct value of the endogenous control, β−actin, and the comparative method (2^-∆∆Ct^) was then applied using control group as calibrator.

### Primary cortical cultures

#### Cortical cultures

Cortical cultures were prepared from brains of embryonic day 17–18 (E17/E18) embryos from timed pregnant Wistar rats (Charles River), as previously reported^40^. In brief, cortex was dissected out in Hanks’ balanced salt solution buffered with Hepes and dissociated via trypsin treatment. Cells were plated at 1 × 10^6^ cells on 3.5-cm dishes precoated with poly-L-lysine. After 2 days of culturing in neurobasal medium with B-27 supplement (0.5 mM L-glutamine, 1% antibiotic penicillin/streptomycin), half of the medium was changed every 3–4 days. All experimental treatments were performed on 12-day “*in vitro*” (DIV) cultures in Neurobasal + ½ B27 fresh medium. The culture cell composition was examined using immunocytochemical staining for neurons (NeuN antibody, Sigma, 1: 200), astrocytes (GFAP antibody, Sigma 1:400) and microglia (Iba1 antibody, Abcam 1: 200) with DAPI nuclear staining. Mixed cultures contain about 50% NeuN^+^ cells, 45% GFAP^+^ cells and 4% of Iba1^+^ cells.

#### Hippocampal cultures

Hippocampal cultures were prepared from brain of embryos Wistar rats (Charles River) at embryonic day 17–18 (E17/E18), as previously reported^56^. Briefly, hippocampus was dissected out in Hanks’ balanced salt solution buffered with Hepes and dissociated via trypsin treatment. Cells were plated at 1 × 10^6^ cells on 3.5-cm dishes precoated with poly-L-lysine. After 2 days of culturing in neurobasal medium with B-27 supplement (0.5 mM L-glutamine, 1% antibiotic penicillin/streptomycin), half of the medium was changed every 3–4 days. All experimental treatments were performed on 12-day *in vitro* (DIV) cultures in Neurobasal + ½ B27 fresh medium.

### Cell viability and nuclear morphology

Cell viability was assessed by counting the number of intact nuclei according to the method previously described^57^. Briefly, the culture medium was removed and replaced with 0.5 ml of a detergent containing lysing solution (0.5% ethylhexadecyldimethylammonium bromide, 0.28% acetic acid, 0.5% Triton X-100, 3 mM NaCl, 2 mM MgCl_2_, in phosphate-buffered saline (PBS) pH 7.4 diluted 1/10). After 2 min, cells were collected and the solution consisted of a uniform suspension of single, intact, viable nuclei that were then quantified by counting in hemocytometer since the detergent-containing solution is able to dissolve the nuclei of the cells that are dying, while healthy cells appear as phase-bright intact circles surrounded by a dark ring. Broken or damaged nuclei were not included in the count.

Alternatively, cortical cultures were fixed in 4% paraformaldehyde and permeabilized with 0.2% Triton X-100 in Tris HCl 0.1 M pH 7.4 for 5 min and then incubated with Hoechst 33258 (0.25 μg/ml) for 5 min at room temperature. After washing with PBS, the percentage of shrunken and condensed nuclei was assessed. Apoptotic nuclei were then visualized by a Leica fluorescent photomicroscope and scored by counting 12 microscopic fields per coverslip in 2 coverslips from 4 experiments.

### Western blotting

For cytoplasmic lysates, neurons were washed twice with ice-cold PBS, lysed in lysis buffer (1% NP40, 50 mM Tris-HCl, pH8) and cetrifuged before the addition of 1X protease inhibitor mixture. Protein concentration was measured using a Biorad DC protein assay kit (Bio-Rad) and equivalent amounts of protein (10–30 μg) were separated on 4–12% Bis-Tris SDS-PAGE gels (Invitrogen), blocked with 5% milk for 30 min and then incubated overnight with anti rabbit cleaved caspase 3 (Asp175) (Cell Signaling 1:1000) or anti β-actin (Sigma (1:10000) or monoclonal anti Aβ (6E10, Covance 1:500).

Incubation with anti-rabbit secondary antibodies peroxidase-coupled was performed for 1 h at room temperature. Immunoreactivity was developed with enhanced chemiluminescence (ECL system; Amersham, Arlington Heights, IL) and visualized by autoradiography.

### Immunofluorescence

Living mixed cortical cells were stained for 1 h at 4 °C using rabbit polyclonal anti-PKR_1_ or anti-PKR_2_ antibodies 1:400 in PBS (Alomone Labs, Jerusalem, Israel), washed in PBS and incubated with a goat anti-rabbit rhodamine-conjugated secondary antibody (Sigma) for 30 min at room temperature. This procedure allowed us to specifically target surface protein expression. Cells were then fixed in 4% (w/v in PBS) paraformaldehyde for 10 min at room temperature on immunofluorescence-labeled coverslips. For PROK2 immunofluorescence, CNs were first fixed in 4% (w/v in PBS) paraformaldehyde following the above procedure, incubated with rabbit polyclonal anti-PROK2 antibody (Abcam, Cambridge, UK 1:400), washed in PBS and incubated with a goat anti-rabbit rhodamine-conjugated secondary antibody (Sigma) for 30 min at room temperature.

CNs cells were then incubated with mouse anti-NeuN (Sigma, 1:200 dilution) or mouse anti-GFAP (Sigma, 1:400 dilution) overnight at 4 °C and with a goat anti-mouse alexa-488 conjugated secondary antibody (Sigma, 1:400) for 30 min at room temperature. For nuclei visualization, coverslips were incubated with Hoecsht 33258 (0,25 μg/ml) for 5 min at room temperature. Cells were visualized by a confocal laser scanning microscope (Leica SP5, Leica Microsystems, Wetzlar, Germany). Final figures were assembled by using Adobe Photoshop 7 and Adobe Illustrator 10.

### Extracellular recordings

Electrophysiological recordings were performed in male Tg2576 transgenic mice aged 6–9 months using standard procedures^58^. Tg2576 mice and their non-transgenic littermates were purchased from Taconic Europe (Lille Skensved, Denmark). Preparation of hippocampal slices was performed in accordance with the European Communities Council Directive (86/609/EEC). Vibratome-cut parasagittal slices (400 μm) were prepared, incubated for 1 h and then transferred to a recording chamber submerged in a continuously flowing artificial CSF (30 °C, 2–3 ml/min) gassed with 95% O_2_ and 5% CO_2_. The composition of the control solution was (in mM): 126 NaCl, 2.5 KCl, 1.2 MgCl_2_, 1.2 NaH_2_PO_4_, 2.4 CaCl_2_, 11 glucose, 25 NaHCO_3_. fEPSPs were recorded in the *stratum radiatum* of the CA1 using glass microelectrodes (1–5 MΩ) filled with artificial CSF. Paired pulse facilitation (PPF) was assessed at inter-stimulus intervals ranging from 20 to 500 ms. An additional 30 min baseline period was obtained before attempting to induce long-term potentiation (LTP). LTP was induced by a high- frequency stimulation (HFS) protocol (1 train, 100 Hz, 1 s) and the effect of conditioning train was expressed as the mean (±SEM) percentage of baseline EPSP slopes measured at 60 min after stimulation protocol. Statistical analysis was evaluated by unpaired Student’s t test (significance was set at p < 0.05).

### Data analysis

Statistical analysis was performed using SPSS 11.0.0 for Windows (SPSS Inc., USA). All results are expressed as mean + SEM, with n the number of independent experiments. The significance of the effect was performed by one-way analysis of variance (ANOVA) followed by Bonferroni’s test for multiple comparisons. The significance level was set at p < 0.05 (*) and p < 0.01 (**).

## Additional Information

**How to cite this article**: Cinzia, S. *et al.* Bv8/prokineticin 2 is involved in Aβ-induced neurotoxicity. *Sci. Rep.* **5**, 15301; doi: 10.1038/srep15301 (2015).

## Supplementary Material

Supplementary fig.1

Supplementary fig.2

Supplementary fig.3
