# Supplementary material for: Bv8/prokineticin 2 is involved in Aβ-induced neurotoxicity
Source: Sci Rep. 2015 Oct 19;5:15301. doi: 10.1038/srep15301 (PMC4610025; doi:10.1038/srep15301)
Supplement: Supplementary fig.1 [file srep15301-s1.pdf]

Bv8/prokineticin 2 is involved in Aβ-induced neurotoxicity by Severini Cinzia Lattanzi Roberta, Maftei Daniela, Marconi Veronica, Ciotti Maria Teresa, Petrocchi Passeri Pamela, Florenzano Fulvio, Del Duca Ester, Caioli Silvia, Zona Cristina, Balboni Gianfranco, Salvadori Severo, Nisticò Robert, Negri Lucia

Additional Figure 1

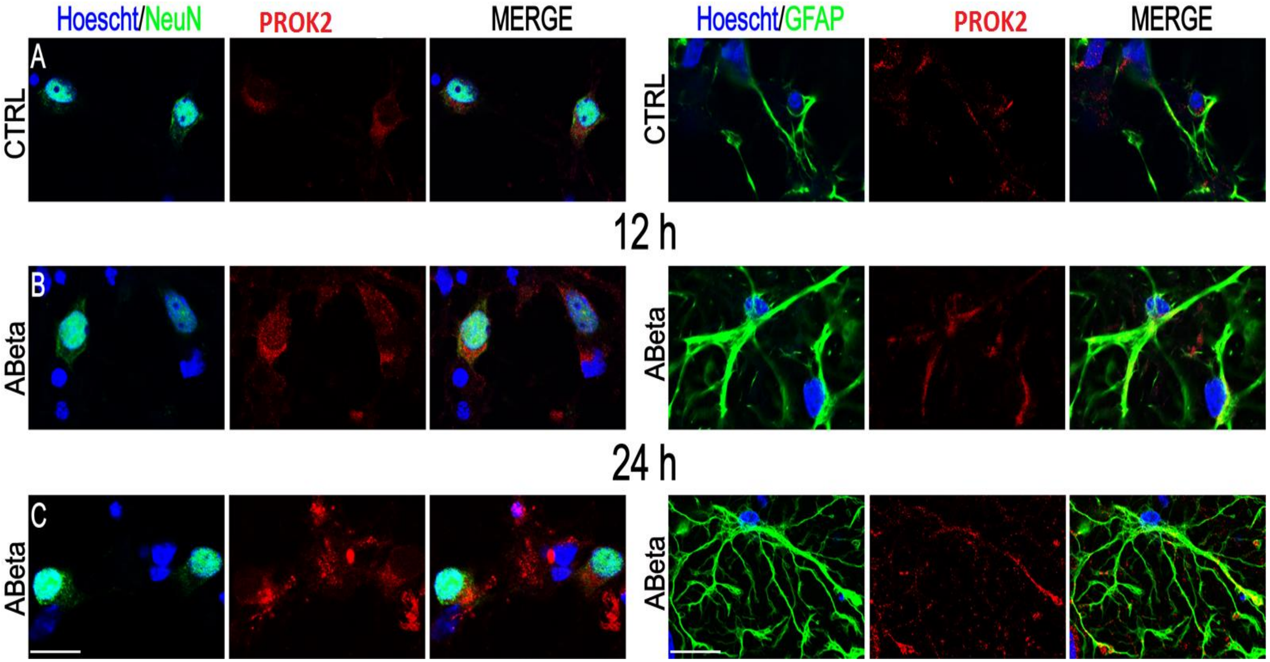

PROK2 protein localization in control conditions and after 12 and 24h $A\beta_{1-42}$ treatment
Representative confocal images of cultured mixed cortical neurons (CNs) after 12 and 24 hours $A\beta_{1-42}$ treatment (ABeta) stained for PROK2, a neuronal marker (NeuN, green) or an astrocytes marker (GFAP, green). Nuclei were counterstained with Hoecsht (blue). **A**) In control conditions, low to medium intensity levels of PROK2 immunoreactivity were detected, mainly located in the neuronal bodies cytoplasm (left row) or in the astrocytes processes (right row). By zooming in the images, to observe the PROK2 intracellular distribution pattern, it was evident a rather homogeneous distribution, characterized by immunopositive puncta in both neurons and astrocytes. **B**) After 12 hours $A\beta_{1-42}$ treatment, PROK2 immunoreactivity showed a clear, although moderate intensity levels increase in both neurons and astrocytes. **C**) After 24 hours $A\beta_{1-42}$ treatment, PROK2 immunoreactivity intensity levels further increased in both neurons and astrocytes. Several PROK2 immunopositive puncta were still detectable, although dense clusters and aggregates were very diffuse, in particular in neurons and astrocytic processes, showing degenerative features. Scale bar: 15 μm.
