# Supplementary material for: Bv8/prokineticin 2 is involved in Aβ-induced neurotoxicity
Source: Sci Rep. 2015 Oct 19;5:15301. doi: 10.1038/srep15301 (PMC4610025; doi:10.1038/srep15301)
Supplement: Supplementary fig.2 [file srep15301-s2.pdf]

Bv8/prokineticin 2 is involved in Aβ-induced neurotoxicity by Severini Cinzia Lattanzi Roberta, Maftei Daniela, Marconi Veronica, Ciotti Maria Teresa, Petrocchi Passeri Pamela, Florenzano Fulvio, Del Duca Ester, Caioli Silvia, Zona Cristina, Balboni Gianfranco, Salvadori Severo, Nisticò Robert, Negri Lucia

Additional Figure 2

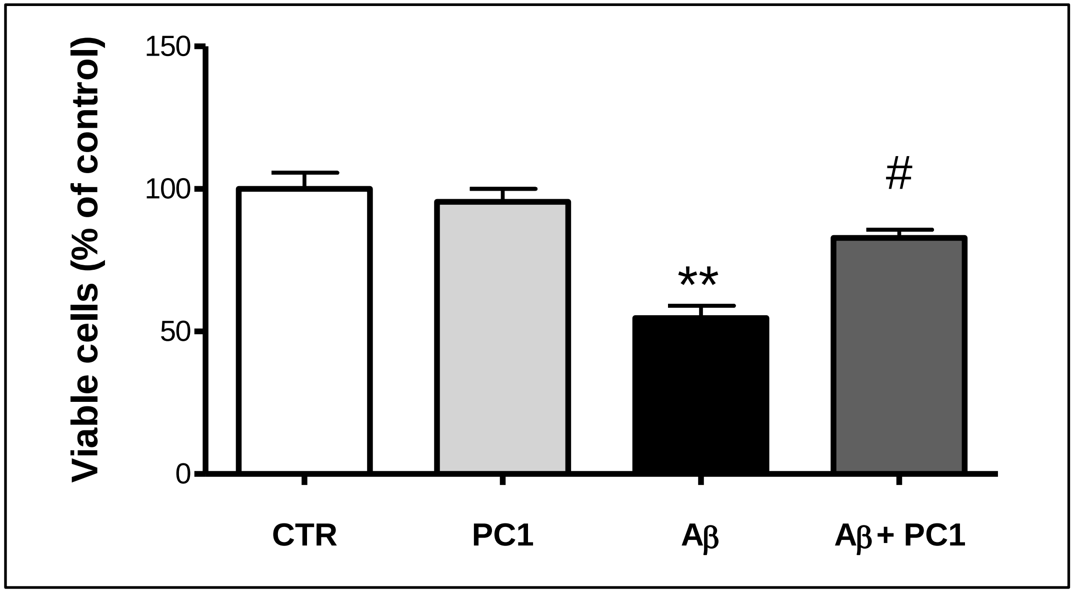

Effect of PC1 against Aβ neurotoxicity in hippocampal cultures (HNs)
HNs (1×10^6 cells/well) were treated at 12 DIV with $A\beta_{1\text{-}42}$ (20μM) alone or in the presence of PC1 (100nM) and then assayed for cell viability 48h later. Data from PC1 alone are also shown. Data represent mean (±SEM) from 3 independent experiments run in duplicate. Statistically significant differences were calculated by one-way analysis of variance (ANOVA) for repeated measures followed by Bonferroni's test for multiple comparisons (**p<0.01 vs CTR, #p<0.01 versus $A\beta_{1\text{-}42}$).
