# Supplementary material for: Bv8/prokineticin 2 is involved in Aβ-induced neurotoxicity
Source: Sci Rep. 2015 Oct 19;5:15301. doi: 10.1038/srep15301 (PMC4610025; doi:10.1038/srep15301)
Supplement: Supplementary fig.3 [file srep15301-s3.pdf]

Bv8/prokineticin 2 is involved in Aβ-induced neurotoxicity by Severini Cinzia Lattanzi Roberta, Maftei Daniela, Marconi Veronica, Ciotti Maria Teresa, Petrocchi Passeri Pamela, Florenzano Fulvio, Del Duca Ester, Caioli Silvia, Zona Cristina, Balboni Gianfranco, Salvadori Severo, Nisticò Robert, Negri Lucia

Additional Figure 3

$A\beta_{1\text{-}42}$ aggregation

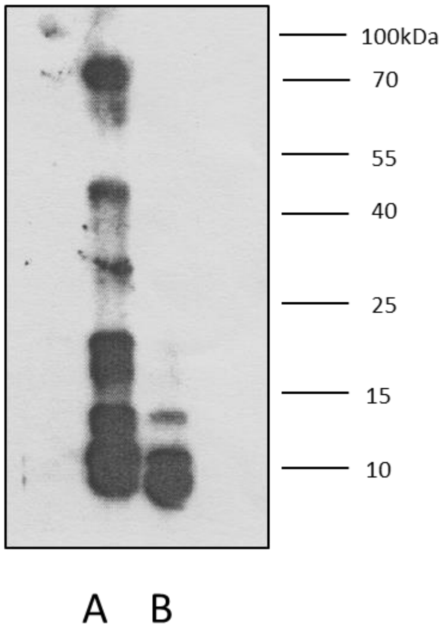

Western blot analysis of **A**) aggregated and **B**) non-aggregated $A\beta_{1\text{-}42}$. Aliquots of in $A\beta_{1\text{-}42}$ (5μM in PBS) were incubated for 72h at 37°C. Loading buffer was added to equal peptide amounts and separated by 4-12% tris-tricine SDS-PAGE alongside a non-aggregated control. Immunoblotting with 6E10 antibody labeled monomers, dimers, as well as high molecular aggregates.

A= aggregated form (72h)
B= non-aggregated form
